# Linking gut microbiota rhythmicity to circadian maturation in infants

**DOI:** 10.1038/s41598-025-25424-3

**Published:** 2025-11-24

**Authors:** Christophe Mühlematter, Dennis S. Nielsen, Josue L. Castro-Mejía, Jean-Claude Walser, Sarah F. Schoch, Salome Kurth

**Affiliations:** 1 Department of Psychology, University of Fribourg, Rue P.-A.-de-Faucigny 2, CH-1700 Fribourg, Switzerland; 2 Department of Food Science, Faculty of Science, University of Copenhagen, Copenhagen, Denmark; 3 Genetic Diversity Center, ETH Zurich, Zurich, Switzerland; 4 Child Development Center, University Childrens Hospital Zurich, Zurich, Switzerland; 5 Department of Pulmonology, University Hospital Zurich, Zurich, Switzerland

**Keywords:** Actimetry, Circadian development, Host-microbiome interaction, Sleep regulation, Pediatric research, Longitudinal, Paediatric research, Predictive markers

## Abstract

The human gut microbiota undergoes daily fluctuations, yet its interaction with sleep-wake patterns during infancy remains largely uncharted. This study aims to elucidate the relationship between gut microbiota rhythmicity and the development of sleep patterns in infants over the first year of life. We continuously monitored 162 healthy infants across multiple days at 3, 6, and 12 months of age using ankle actigraphy and 24-hour diaries. The Circadian Function Index (CFI) was computed as a proxy for sleep-wake rhythm maturation. Stool samples were collected to profile gut microbiota taxa composition via 16 S rRNA gene amplicon sequencing, and microbial oscillations were assessed through sine and cosine fitting to detect 24-hour patterns. Our findings revealed that the relative abundance of bacterial taxa exhibited rhythmic patterns, with 26 zOTUs (1.74%) following a sine pattern and 100 zOTUs (6.69%) displaying cosine rhythmicity. Cosine rhythmicity became more pronounced with age, showing strong maturation: 7 zOTUs at 3 months, 2 zOTUs at 6 months, and 86 zOTUs at 12 months. Notably, 105 zOTUs (7.02%) were associated with CFI, demonstrating a significant relationship between gut microbiota rhythms and sleep development. Among these, 27 zOTUs with sine dynamics and 96 zOTUs with cosine dynamics were linked to CFI, with this association strengthening as infants aged. These results highlight the increasing synchronization between gut microbiota rhythmicity and sleep-wake cycles during infancy, pointing to a critical window for potential health interventions. This novel observation, previously reported in rodents and adults, underscores the role of gut microbiota in early human development, offering new avenues for enhancing developmental outcomes through targeted interventions.

## Introduction

Infancy represents a critical developmental phase of the human brain. During early childhood sleep undergoes pronounced maturation, a process essential for cognitive and neurophysiological development^1^. Core electrophysiological features of sleep, such as slow waves and spindles, have been identified as biomarkers for the maturation of neuronal connectivity and function^2,3^. Sleep furthermore affects immuno-regulation^4^, cardiovascular function^5^, and metabolic balance^6^, underscoring the necessity of adequate sleep to both neurological development and physiological health. Early human life is characterized by notable variability in individual sleep behavior^7,8^. Despite the substantial health implications, such as effects on cognitive development and obesity risk (Li et al., 2022; Morales-Muñoz et al., 2021), the underlying drivers of this variability remain largely obscure. The complexity of capturing sleep maturation through continuous monitoring presents a substantial challenge, hindering a thorough understanding of infant sleep patterns. During infancy, neurophysiology and behavior are highly modifiable, allowing for effective re-shaping of developmental trajectories through timely interventions^9^. For example, early sleep interventions have been shown to extend nighttime sleep duration, reduce night wakings, and promote self-soothing behaviors, all of which contribute to improved sleep quality and support healthy development. This highlights a promising approach to enhancing sleep regulation and fostering positive developmental outcomes.

Given the multifaceted factors influencing sleep, recent research has uncovered a bidirectional relationship between sleep and gut microbiota—the microorganisms inhabiting the gastrointestinal tract^10^. Modifying the gut microbiota through antibiotics can disrupt sleep patterns, such as reducing non-rapid eye movement sleep (NREMS) and increasing REM sleep episodes in mice, or decreasing slow-wave sleep (SWS) in humans and rats, suggesting a role for microbiota in maintaining healthy sleep architecture^11–13^. Conversely, sleep disruptions have been found to alter gut microbiota composition^14,15^. In adults, a fraction of variability in sleep quality and duration is explained by gut microbiota profiles, underscoring the interconnectedness of sleep to microbial signatures^16,17^. The sleep-gut connection is not limited to adulthood, but is observed in adolescence, childhood, and infancy; likely it primes health outcomes across the lifespan^18–21^.

Similar to sleep, variability in individual gut microbiota during infancy is large^22^, and defines critical health aspects such as metabolism^23^, immune function^24^, and neurodevelopment^25^. Colonization of the infant gut begins at birth, and across the first three years of life microbial diversity, stability, and complexity increase. The succession of microbial communities in a healthy infant gut is influenced by environmental and dietary factors, such as mode of birth, antibiotic usage, and dietary habits^26–28^. Furthermore, gut microbiota have recently been found to undergo “diurnal” dynamics, demonstrating variance in abundance and function across the day^15,29^. Animal models show that diurnal microbial dynamics are tied to the host’s circadian rhythm, as bacterial diurnal variation is partially regulated by their host’s clock genes (Per1/2, Bmal1)^30,31^. However, this interaction between the host’s circadian rhythm and bacterial rhythm appears to be bidirectional: in germ-free mice, the absence of microorganisms disrupts the intestinal epithelial cell clock, decreasing the expression of core clock genes such as Bmal1 and Cry1, while increasing Per1 and Per2. This disruption alters metabolic homeostasis and rhythmic expression in the intestinal cells^32^. Further evidence supporting the role of the gut microbiome as a “bottom-up” regulator of the circadian clock is provided by observations that microbial metabolites modulate the peripheral clock in mice^33^. This suggests that the interaction between the gut microbiota and the host’s biological clock extends beyond mere temporal coordination but also includes but also includes regulatory effects on the host’s circadian clock function.

In addition to these observations in adult animals, novel studies suggest that diurnal variations also occur in the gut microbiota of infants^34^. However, their connection to the infants’ circadian rhythm remains unknown. Addressing this phenomenon requires longitudinal data across infancy, collected from large, well-controlled cohorts with objective and continuous assessments of sleep. Ankle-actigraphy captures physiological acceleration, thereby providing reliable data that captures the large individual variability in infant rest-activity patterns^35,36^. Applying the Circadian Function Index (CFI)^37^ can serve as a proxy for circadian maturation; facilitating the investigation of its relationship with gut microbiota dynamics.

We hypothesized that individual gut microbiota profiles are associated with the maturation of infant circadian rhythm, using an increased CFI as proxy for this maturation. This relationship would indicate that as the circadian rhythm matures, distinct shifts in gut microbiota composition can be observed. Additionally, we predicted a maturation of diurnal rhythm in gut microbiota composition across the first year of life. Specifically, we anticipated that the proportion of rhythmic zOTUs would increase with age. Third, we expected that the diurnal microbial rhythm is linked to infants’ sleep regulation, quantified by the CFI. A linkage would indicate that as the circadian rhythm develops and strengthens, the rhythmic expression of the gut microbiota also becomes more pronounced.

## Methods

### Study population

This study was conducted in Switzerland and included 162 healthy infants assessed at ages 3–12 months from 2016 to 2019^19^. Infants included in the study were term-born, showed no signs of acute or chronic illness, were delivered vaginally, and received at least 50% of their nutrition from breast milk at the time of enrollment. Exclusion criteria encompassed the use of antibiotics or sleep-affecting medications, chronic illnesses, or disorders of the central nervous system. The cantonal ethics committee of Zurich, Switzerland, approved the project (BASEC 2016−00730), in line with the principles of the Helsinki Declaration. Before study enrollment, parents received detailed explanations about the study procedures and then provided written informed consent for study participation.

## Study design

Families and infants were longitudinal assessed at 3, 6, and 12 months of age. For each assessment, actigraphy data was collected for 11 consecutive days (9.16 ± 1.23 days) using a GENEactiv sensor (Activinsights LtD, Kimbolton, UK, 43 × 40 × 13 mm, MEMS sensor, 16 g, 30 Hz frequency) attached to the infant’s left ankle (either with a paper strap or in a modified sock). Concurrently, caregivers completed a 24 h paper-pencil sleep diary to document sleep and wake periods, any temporary removal of the device, and external movements during sleep (e.g., such as when the infant was in a moving vehicle)^36,38^. Additionally, caregivers collected at least one infant stool sample during each assessment, for which clock time was documented. Demographic data and complementary information was collected with online questionnaires at each assessment timepoint (SoSci Survey;^39^.

## Data processing

Actimetric activity counts were processed in Matlab (R2016b), by using binary files extracted through GENEactiv PC Software (version 3.1). Transformation involved applying a 3–11 Hz bandpass filter and compressing the signal into 15-second bins. Acceleration data from all three axes was combined using a sum of squares. In all cases, actigraphy data were analyzed in combination with concurrent parent-completed 24-hour sleep diaries, following established laboratory procedures^38^. Data were systematically cross-checked, using the diaries to annotate periods of device non-wear, external movement (e.g., stroller, car rides), or discrepancies between actimetry and diary records, in accordance with best practices in pediatric actigraphy^40^. The acceleration signal was compressed to one data point per minute through data summation^8^. This compressed acceleration data was then used to compute the CFI as a proxy for circadian maturation.

## Circadian function index (CFI)

We selected the CFI due to its suitability as a non-invasive and continuous marker of circadian sleep-wake development, capturing both, intra- and inter-daily rhythms dynamics. While previous studies used infant ankle actimetry to assess sleep quantity and continuity^8,35^, the use of CFI captures the maturation of sleep-wake rhythmicity across several days, introducing a novel perspective for pediatric circadian development. CFI measures the regularity of activity-rest patterns with a score from 0 to 1^37^. The computation of CFI relies on 3 measures, computed through the R package “nparact” version 0.8^41^: Intradaily Variability (IV), Interdaily Stability (IS), and Relative Amplitude (RA). IV determines the frequency and extent of transitions between rest and activity within a 24-hour day, such that a lower IV represents more consolidated rest-activity periods, whereas a higher IV suggests frequent transitions and thus fragmented sleep patterns. IS quantifies the similarity of activity patterns across days. A higher IS represents a more stable day-to-day activity-rest pattern. RA measures the amplitude between the most active and least active periods within a 24-hour cycle. A higher RA indicates a more pronounced difference between peak activity and rest periods.

For each infant, these three scores were averaged across days of continuous actigraphy data collection, covering an average period of 9.16 ± 1.23 days across all ages (3, 6, and 12 months). Specifically, at 3 months, infants were assessed over an average of 9.28 ± 1.17 days. At 6 months, the average was 9.14 ± 1.46 days. By 12 months, the average assessment period was 9.04 ± 0.99 days. The CFI was then calculated with the following formula:

$${\text{CFI}} = \frac{{({\text{IS}} + (2 - {\text{IV}})/2 + {\text{RA}})}}{3}$$

This formula combines each component to provide an overall measure of circadian maturation, with higher CFI values reflecting more regular and stable activity-rest patterns.

## DNA extraction from stool and processing of gut microbiota

Gut microbiota profiles were computed from stool samples collected at infant ages 3, 6, and 12 months. Upon collection, samples were stored in the participants’ fridge (approximately 4 °C) before being transported at a consistent temperature within 96 h to the laboratory. Upon arrival, samples were divided into 200 mg aliquots and preserved at -50 °C until processing. Total DNA extraction was performed using a PowerSoil DNA Isolation Kit (MOBIO Laboratories, Carlsbad, CA, USA), following the manufacturer’s guidelines with small modifications (for details see^19^. 16 S rRNA gene amplicon libraries were prepared using specific primers targeting the V3 region^42^, equipped with adapters compatible with the Nextera Index Kit^®^ (Illumina, CA, USA): NXt_338_F: 5′- TCG TCG GCA GCG TCA GAT GTG TAT AAG AGA CAG ACW CCT ACG GGW GGC AGC AG -3′ and NXt_518_R: 5′- GTC TCG TGG GCT CGG AGA TGT GTA TAA GAG ACA GAT TAC CGC GGC TGC TGG − 3′. Subsequent steps for amplification profiling, barcoding, purification of amplicon libraries, and sequencing were carried out as previously described^43^. Initial quality assessment of the raw datasets (2 × 151 bp, paired-end) was conducted using FastQC58. Overlapping reverse and forward reads were trimmed using SeqKit59, and reads were merged with FLASH (v1.2.11)^44^, allowing for overlaps between 15 and 300, with a maximum mismatch density of 0.25. Cutadapt (v1.12)^45^ was utilized for primer region trimming, maintaining an error rate below 0.01. Prinseq^46^ was used for quality filtering by discarding reads containing ambiguous nucleotides or with an average quality score below 20. Zero-radius Operational Taxonomic Units (zOTUs) were identified using usearch (UNOISE3 v10.0.240)^47^, and taxonomic classifications were determined based on the Greengenes database^48^.

To mitigate batch effects during stool sample processing, batch run was incorporated as a control variable in all statistical models. Samples with fewer than 50,000 reads were excluded from the study (*n* = 6). The remaining samples were rarefied to the lowest observed read count of 50,268. This process resulted in 1430 amplicon sequencing variants. Autoclaved water samples, subjected to the same experimental procedures, served as negative controls and exhibited read counts ranging from 2 to 150,785. zOTU2 (Enterobacteriaceae), identified in 30% of the negative controls and 10% of the stool samples, showed no significant impact on the studied gut microbiota markers. Only bacterial taxa present in at least 20% of the samples from all collection points (3-, 6-, and 12-months) and accounting for at least 1% of total bacterial counts were included in subsequent analyses. This selection process resulted in 1451 zOTUs included in the analysis.

### Rhythmicity analysis

To assess diurnal rhythmicity in the relative abundance of specific bacterial taxa (zOTUs), we adapted a cosinor analysis approach. Cosinor analysis utilizes sinusoidal models to capture periodicity in time-series data and is thus particularly well-suited for biological rhythms^49^. The cosinor approach is well suited for short and sparse data series, providing both rhythm detection and circadian parameter estimation, making it especially useful for real-world data and population-level research^50^. This analysis conventionally employs a cosine function to fit rhythmicity, allowing for direct derivation of the acrophase (i.e., peak timing), a parameter with clear biological interpretation. While both cosine and sine functions mathematically capture the same periodic patterns, cosine fitting offers a more intuitive understanding of phase relationships in biological rhythms^51^. We transformed the clock times of stool sample collection into numerical values representing the time of day, and then applied sine and cosine transformations to these values. By fitting sine and cosine functions, we could evaluate whether zOTU relative abundance exhibited patterns conforming to a 24-hour cycle. This method allowed us to detect and quantify cyclical variations in the relative abundance of microbial populations.

### Statistical analysis

Data processing, modeling, visualization, and statistical analysis was performed using R version 4.2.1 and packages ggplot2^52^, phyloseq^53^, and lme4^54^. We applied linear mixed-effects models to compute associations between the relative abundance of zOTUs (dependent variable) and CFI, clock time of stool sampling (fitted to sine and cosine functions), and age group, allowing us to examine microbial rhythmicity. Microbial rhythmicity was assessed by modeling sine and cosine terms separately for each zOTU, and a zOTU was considered rhythmic if either the sine or cosine term showed a significant association (FDR-corrected p-value < 0.05) with relative abundance at a given age. We further incorporated interactions between sine and cosine fits with CFI to assess the combined effects of infant circadian rhythm and microbial zOTU rhythmicity. For rhythmic zOTUs, we reported the relative proportions of phyla. Control variables in the models included sex (0 for male, 1 for female), breastfeeding status (0 for not or rarely breastfed, 1 for occasionally, regularly, or daily breastfeeding) and sequencing run (categorical, with levels 1 to 5). Random intercepts for each participant were included to capture individual-level variability. The alpha level was set to *P* < 0.05, and to address multiple comparison concerns, we applied False Discovery Rate (FDR) correction to the p-values.

## Results

Key demographic variables including antibiotic use, feeding method (percentage of breastmilk, formula, solids), and age at solid food introduction were summarized for each assessment point (3, 6, and 12 months, Table 1).

**Table 1:** Participant demographics at 3, 6, and 12 months, including antibiotic use, feeding type, and age at solid food introduction.

	3 Months	6 Months	12 Months
Antibiotic use			
Never	100%	97.2%	92.1%
Reported intake	0%	2.8%	7.9%
Feeding type			
Exclusive breastfeeding	86.6%	56.6%	25.0%
Exclusive formula feeding	0%	6.2%	52.1%
Mixed feeding	13.4%	36.6%	11.4%
Age at solid food introduction (months)	4.3 ± 0.6		

**Fig. 1:**
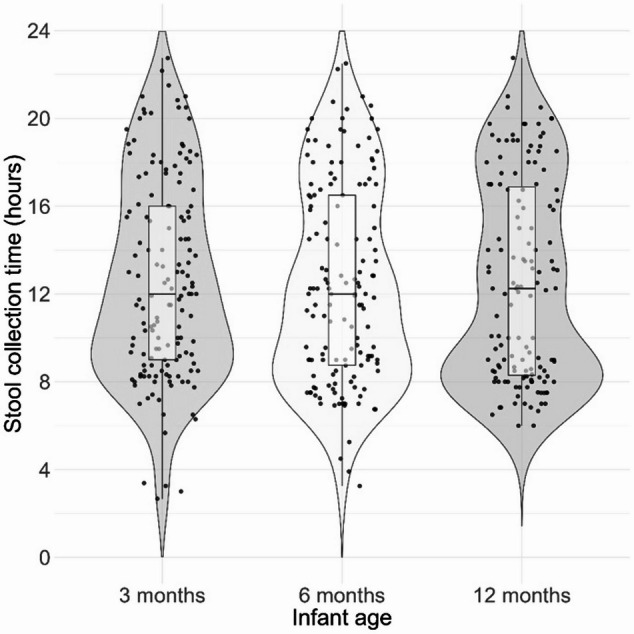
Timing of stool sample collection at each timepoint. Violin and scatter plots depict the distribution of collection times (in decimal hours) at 3, 6, and 12 months. Each dot represents one sample. No significant differences were observed between timepoints (Kruskal-Wallis test, *p* = 0.75).

To assess whether stool sampling times were consistent across participants and age groups, we tested for potential differences in the timing of stool collection across timepoints (Fig. 1), which was not the case (Kruskal-Wallis test, *p* = 0.75; all pairwise Wilcoxon tests *p* = 1 after Bonferroni correction).

### Circadian maturation across infancy

**Fig. 2:**
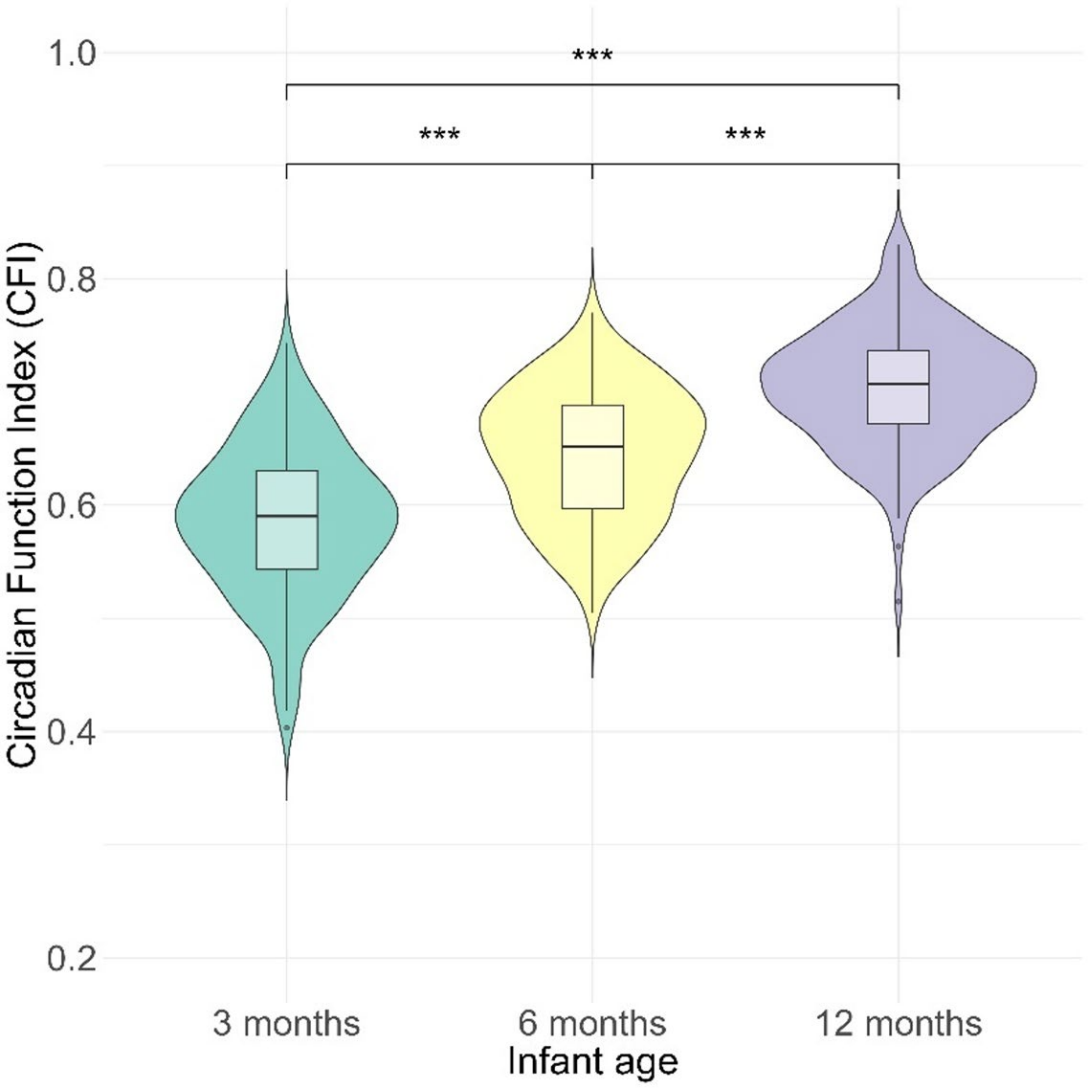
Age-related increase in sleep regulation across infancy assessed with Circadian Function Index (CFI) (Tukey post-hoc tests, *** *p* < 0.001). Central horizontal lines refer to median, the box spans the interquartile range (IQR), covering the middle 50% of data from the 25th to the 75th percentile. Whiskers extend to the highest and lowest values within 1.5 times the IQR from box edges. Points beyond the whiskers represent outliers.

The CFI adequately captured the maturation of infant sleep-wake rhythmicity across age, aligning with developmental expectations of sleep consolidation over time. Across the first year of life, CFI increased with infant age, with mean CFI values of 0.59 ± 0.07 at 3 months, 0.64 ± 0.06 at 6 months, and 0.70 ± 0.05 at 12 months (Fig. 2). CFI increased significantly from 3 months to 6 months (*p* < 0.001, 95% C.I. = 0.04, 0.08; Tukey post-hoc tests), from 3 months to 12 months (*p* < 0.001, 95% C.I. = 0.10, 0.13), and from 6 months to 12 months (*p* < 0.001, 95% C.I. = 0.042, 0.07).

### Association between circadian maturation and gut microbial taxa

To define whether infant CFI underlies differences in gut microbial taxonomic abundance, we analyzed associations between zOTUs relative abundance and CFI (rhythmicity of zOTU abundance is presented subsequently). This identifies the bacterial taxa linked to the infants’ circadian maturational status. We observed both positive and negative correlations between zOTUs and CFI. A total of 105 zOTUs showed significant associations with CFI, representing 7.02% of all identified zOTUs across samples. The strength of this association increased with age: at age 3 months, only 10 zOTUs were linked to CFI, increasing to 17 zOTUs at 6 months, and 87 zOTUs at 12 months. Thus, over the first year of life, a progressively stronger association between infant gut microbiota and sleep rhythm emerges.

**Fig. 3:**
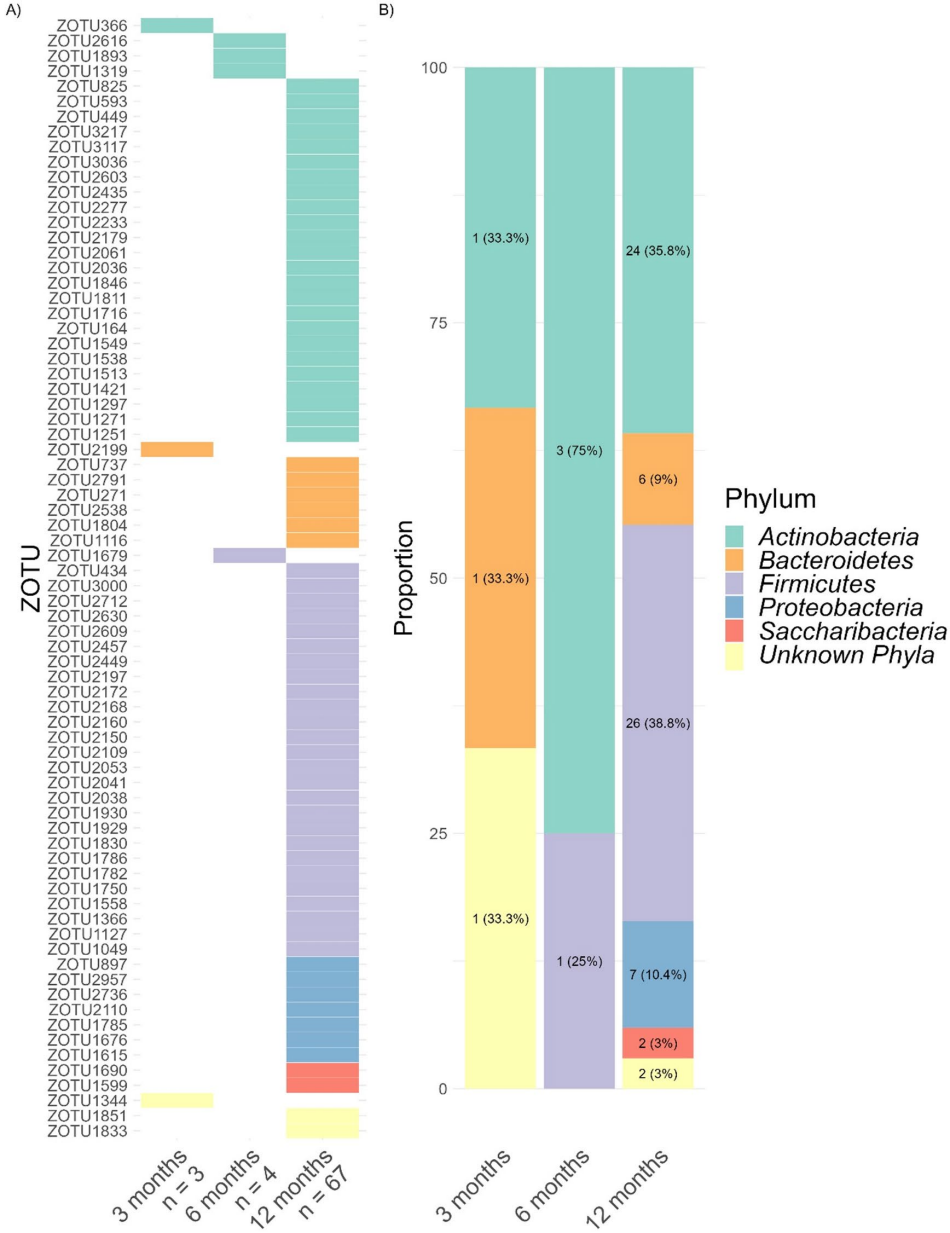
(**A**) Heatmap of zOTUs (*n* = 74) with relative abundance positively associated with infant CFI (*p* < 0.05), specified for each age group. Values below age groups indicate the number of zOTUs. (**B**) Distribution of phyla positively linked to infant CFI.

Among all 105 zOTUs associated with CFI, 74 showed positive correlations (Fig. 3A), indicating that infants with higher CFI had increased relative abundance of these bacterial taxa. At 3 months, among the 3 zOTUs positively correlated with the CFI were 1 Actinobacteria, 1 Bacteroidetes and 1 from unknown phylum (Fig. 3B). At 6 months *Actinobacteria* was the primary phylum associated with CFI, while at 12 months it was mainly *Actinobacteria* and *Firmicutes.*

**Fig. 4:**
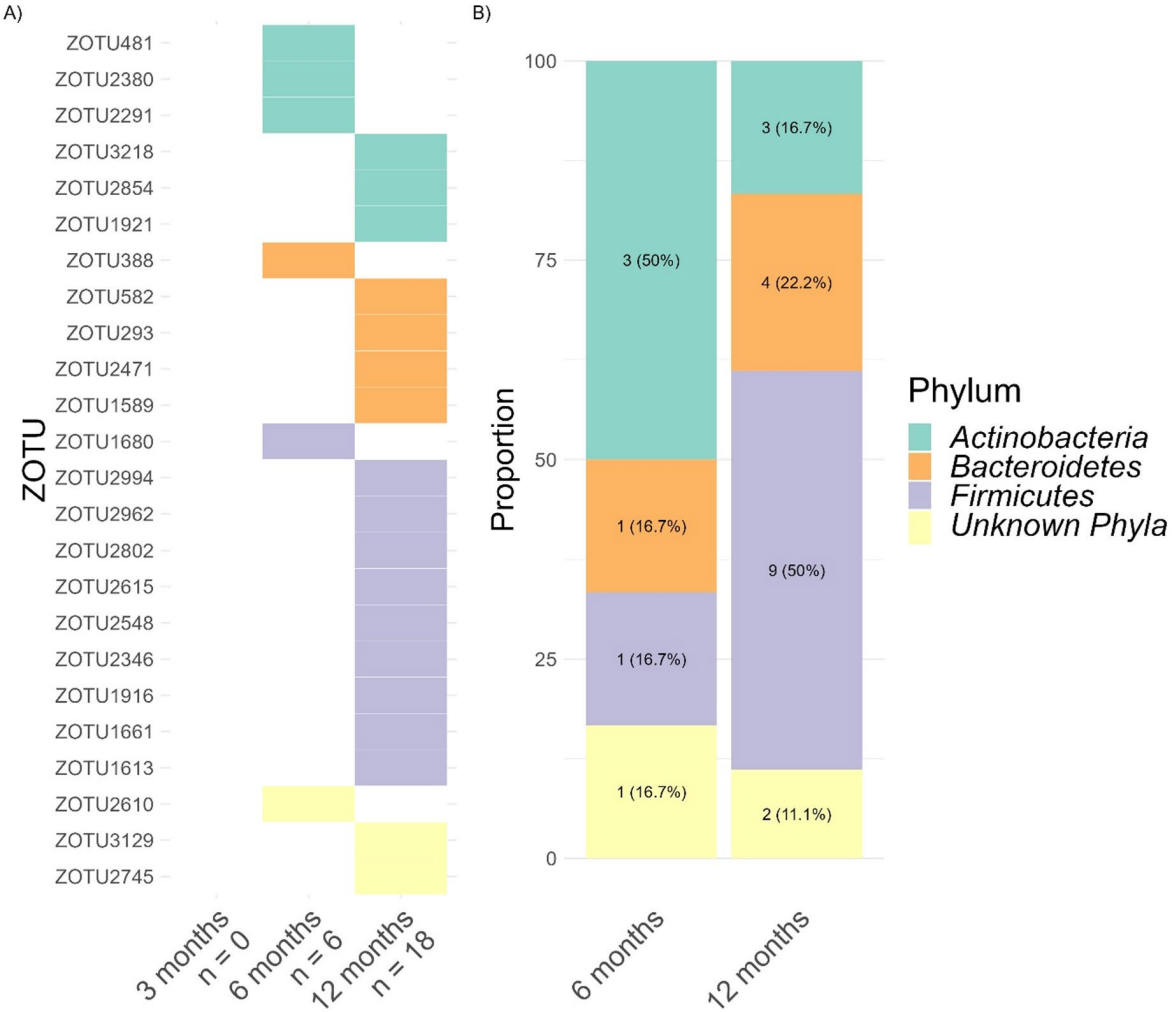
(**A**) Heatmap of zOTUs (*n* = 24) with relative abundance negatively associated with infant CFI (*p* < 0.05), illustrated for each age group. Values below age groups represent the number of associated zOTUs. (**B**) Distribution of zOTUs phyla negatively linked to infant CFI.

24 zOTUs were negatively linked with CFI, indicating that more mature child sleep regulation decreased the relative abundance in these zOTUs (Fig. 4A). No negative association between CFI and gut microbiota relative abundance was observed at 3 months, while it increased to 6 zOTUs at 6 months and 18 zOTUs at 12 months. At 6 months, Actinobacteria was the most frequently associated phylum with CFI, while at 12 months it was Firmicutes (Fig. 4B).

**Fig. 5:**
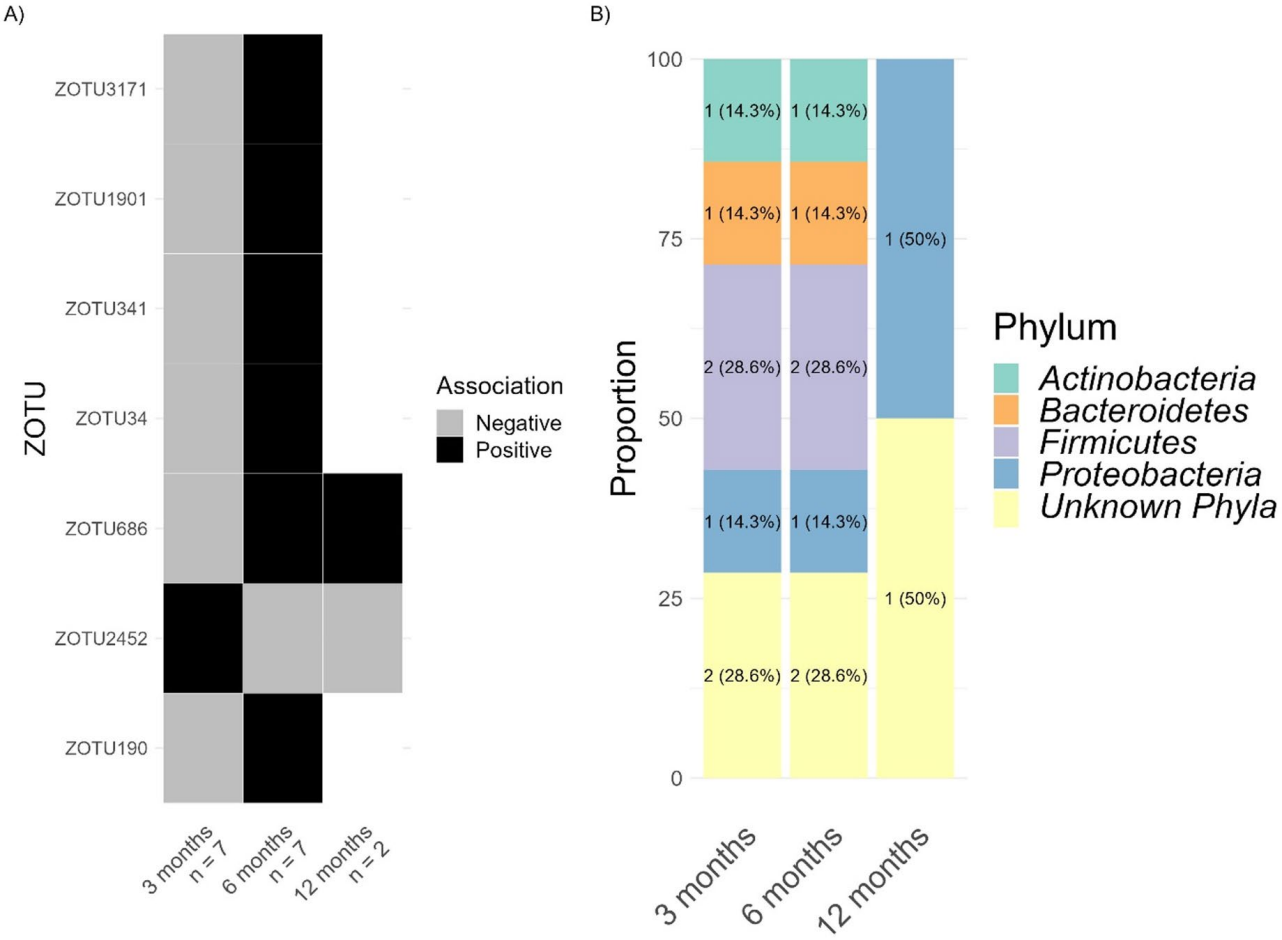
(**A**) Heatmap of zOTUs (*n* = 7) with their relative abundance positively (black) or negatively (grey) associated with infant CFI (*p* < 0.05). Values below age groups represent the number of zOTUs identified. (**B**) Distribution of zOTU’s bacterial phyla linked to infant CFI, showing changes dependent on age.

Interestingly, for 7 zOTUs, the direction of the association between zOTUs relative abundance and CFI was age dependent (Fig. 5A). Specifically, at 3 months 6 zOTUs showed negative associations, which shifted to positive associations at 6 and/or 12 months. Conversely, one zOTU exhibited the opposite: showing positive correlation at 3 months shifting to negative at 6 and 12 months. Among these 7 zOTUs, at 3 and 6 months, there were 2 from the phylum *Firmicutes*, 1 from *Actinobacteria*, 1 from *Bacteroidetes*, 1 from *Proteobacteria* and 2 from unknown phyla. By 12 months, there was 1 zOTU from *Proteobacteria* and 1 from an unknown phylum (Fig. 5B).

### Rhythmic patterns in gut microbiota relative abundance

Subsequently, we investigated whether rhythmicity in gut microbial relative abundance was present during infancy, by considering the influence of clock time of stool sampling. The clock time of stool sampling was fitted to sine and cosine curves capturing potential diurnal effects.

**Fig. 6:**
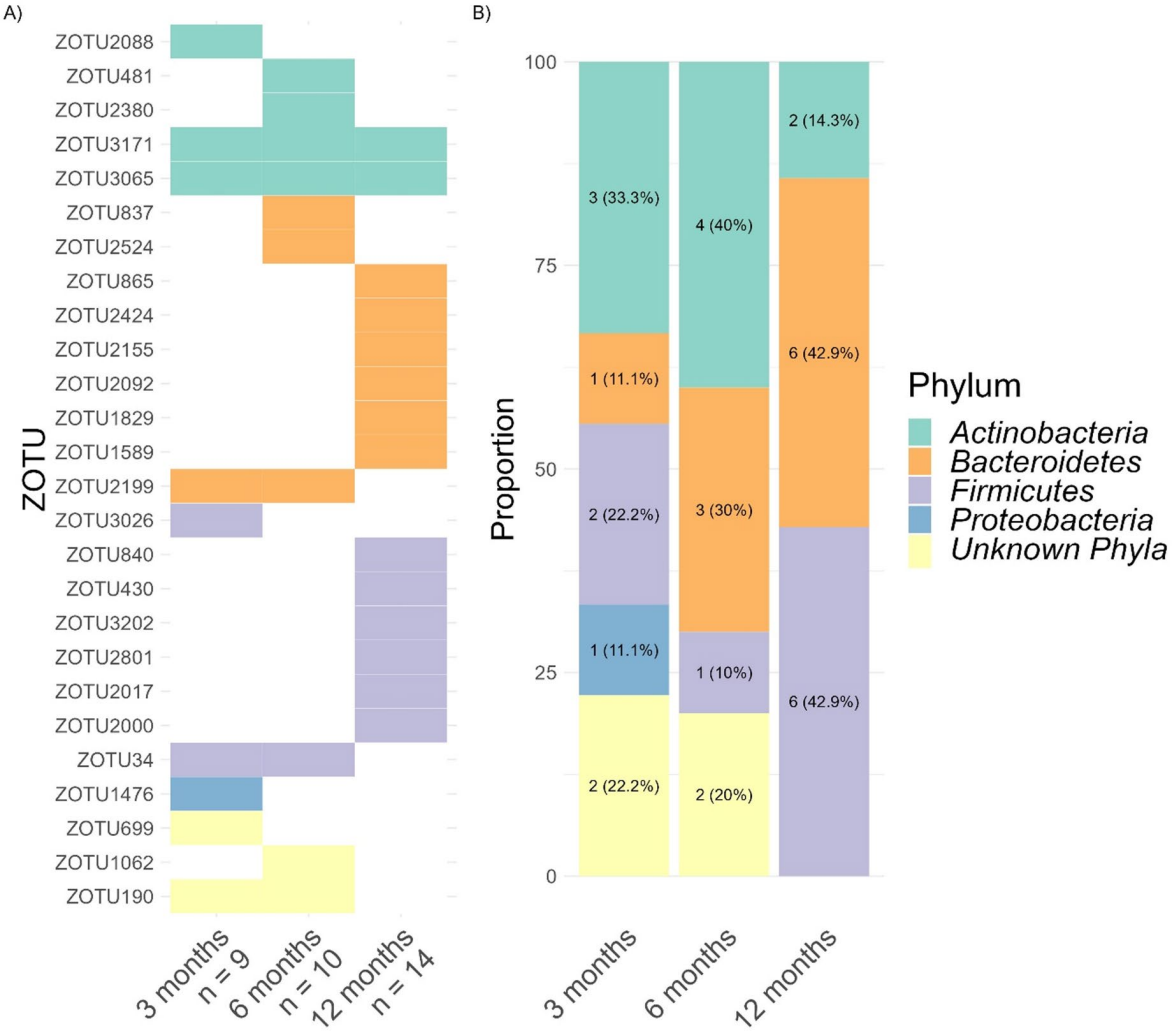
(**A**) Heatmap of zOTUs (*n* = 26) with rhythmic relative abundance following a sine pattern (*p* < 0.05). Values below age groups represent the number of rhythmic zOTUs abundance. (**B**) Distribution of zOTUs phyla following a sine rhythm.

Overall, the relative abundance of 26 zOTUs (i.e., 1.74% of total identified zOTUs) followed a sine pattern, indicating rhythmic diurnal variation in their abundance. The number of rhythmic zOTUs remained relatively constant across age (9 rhythmic zOTUs at 3 month, 10 rhythmic zOTUs at 6 months and 14 rhythmic zOTUs at 12 months; Fig. 6A). *Actinobacteria* was the main rhythmic phyla undergoing a sine pattern at 3 months (33.3%) and 6 months (40%; Fig. 6B). *Bacteroidetes* and *Firmicutes* represented rhythmic phyla at 12 months (each 42.9%).

**Fig. 7:**
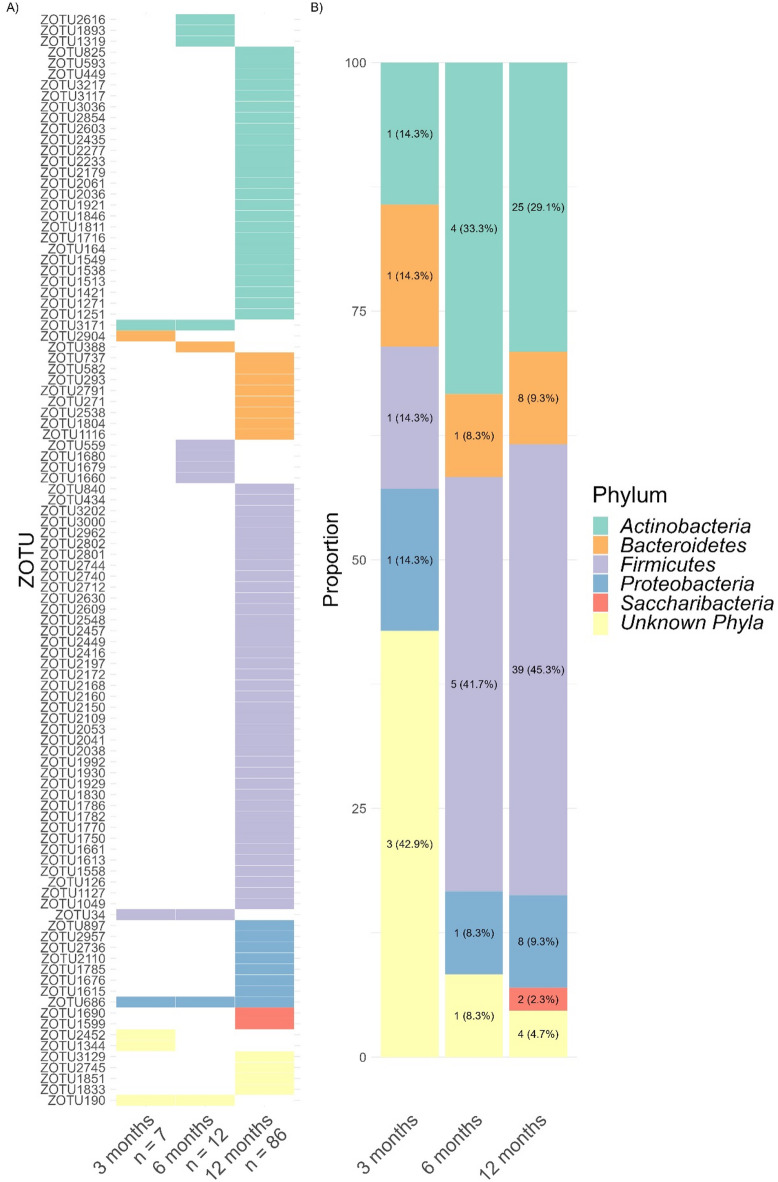
(**A**) Heatmap of zOTUs (*n* = 100) whose relative abundance followed a cosine rhythm (*p* < 0.05). Values below age groups represent the number of zOTUs identified. (**B**) Distribution of zOTU phyla expressed as a cosine pattern.

Next, we applied the cosine approach to zOTU relative abundance. Compared to sine fitting, cosine fitting revealed that a greater number of zOTUs (100 in total, accounting to 6.69% of all zOTUs) exhibited rhythmic dynamics (Fig. 7A). More pronounced than sine, rhythmic cosine dynamics unraveled an age-dependent increase, starting with 7 zOTUs at 3 months, increasing from 12 zOTUs at 6 months to 86 zOTUs at 12 months. The main cosine rhythmic zOTUs bacteria at 3 months were of unknown phylum, while for 6 and 12 months the majority were assigned to *Firmicutes* (respectively 41.7% and 45.3%, Fig. 7B).

### Gut microbiota rhythmicity and infant CFI

**Fig. 8:**
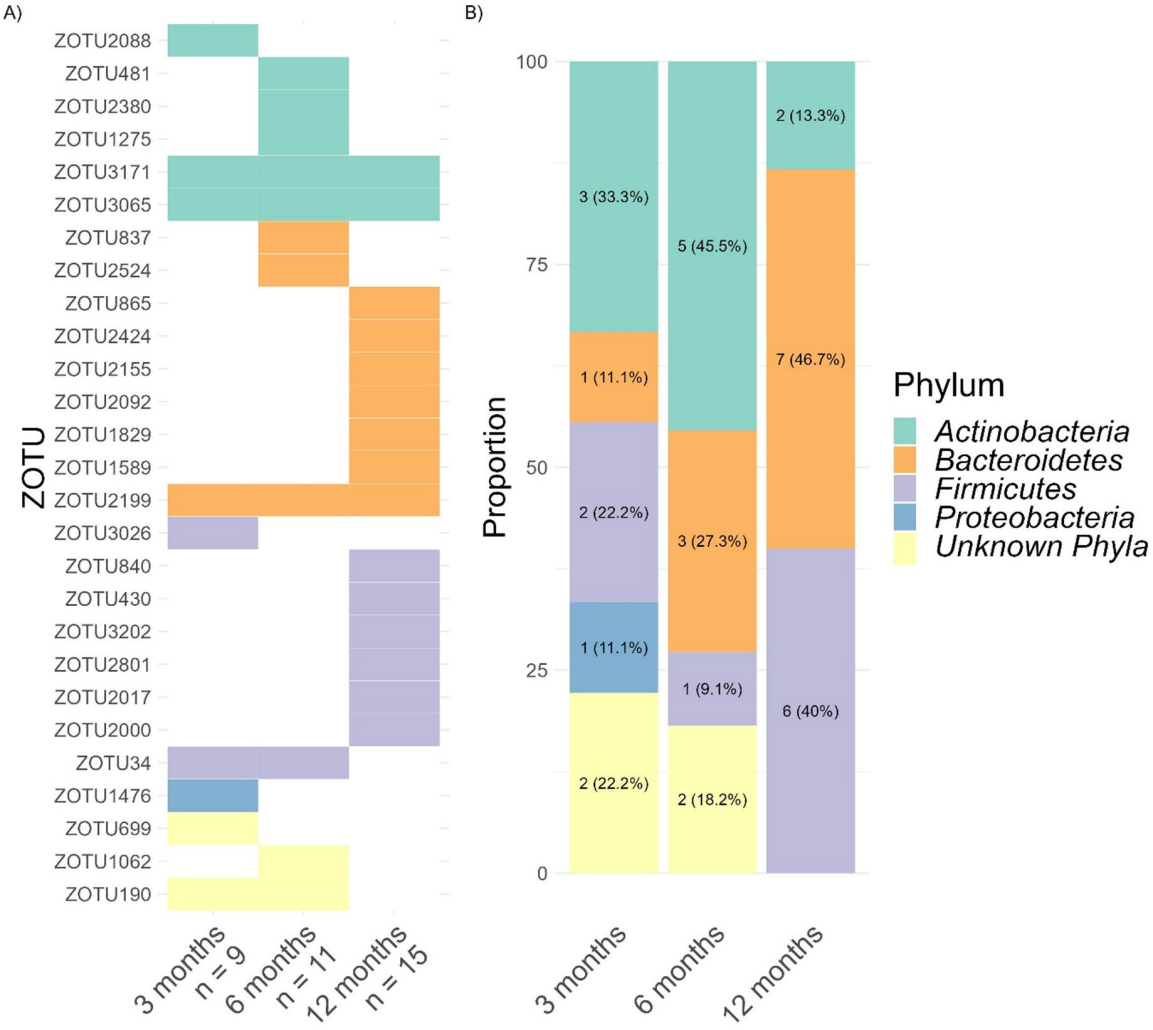
(**A**) Heatmap depicting zOTUs (*n* = 27) with significantly associated sine rhythmicity and CFI (*p* < 0.05). The numbers below age groups indicate the number of zOTUs associated. (**B**) Distribution of zOTUs phyla whose rhythmic sine patterns were linked with CFI.

Finally, we addressed whether gut microbiota rhythmicity relates to infant circadian maturation by testing interactions between zOTU sine and cosine fitting with CFI. Significant interactions would suggest that CFI relates to the rhythmic expression of bacterial composition. Indeed, this interaction was found in sine fitting for 27 zOTUs (1.81% of total zOTUs), with the number of rhythmic zOTUs linked to CFI increasing slightly across 3, 6 and 12 months to 9, 11 and 15 zOTUs, respectively (Fig. 8A). We observed that the interaction of sine rhythmicity with CFI was mainly represented within *Actinobacteria* at 3 and 6 months (33.3% and 45.5% respectively, Fig. 8B), and *Bacteroidetes* at 12 months (46.7%).

**Fig. 9:**
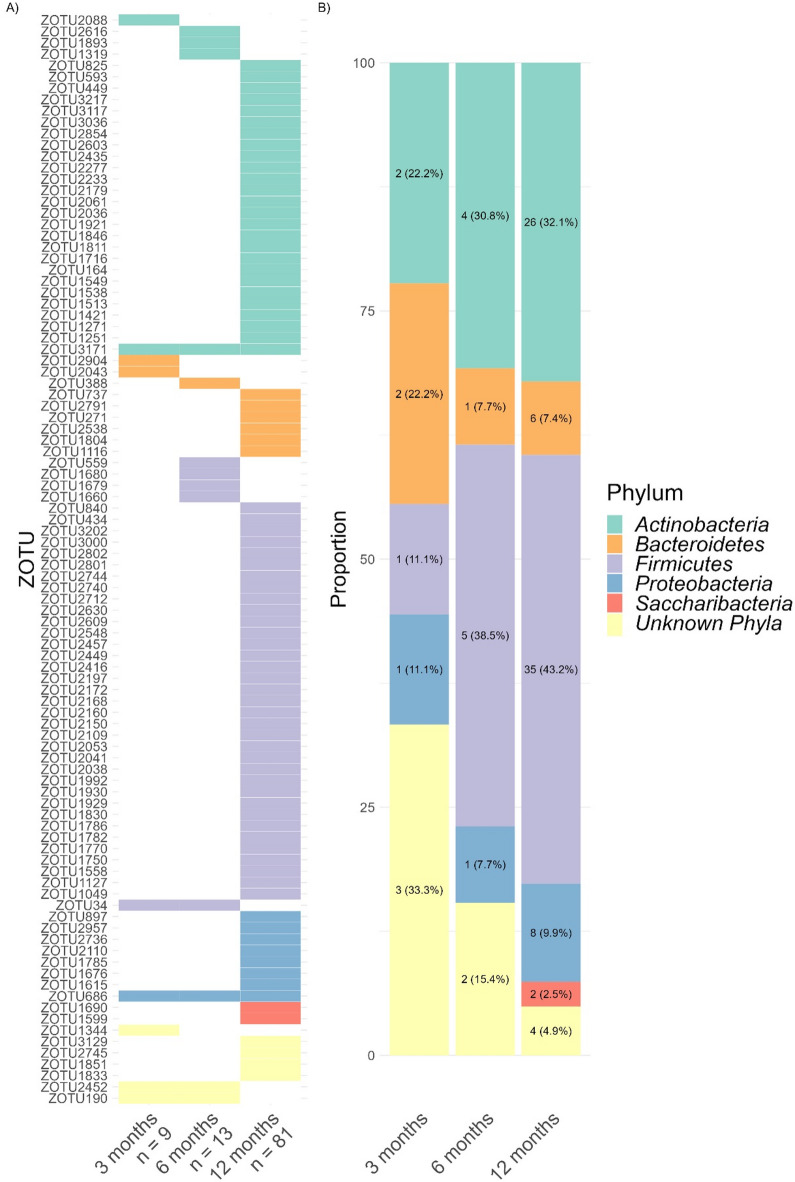
(**A**) Heatmap indicating zOTUs (*n* = 96) with significantly associated cosine rhythm and CFI (*p* < 0.05). Values below age groups represent the number of corresponding zOTUs. (**B**) Distribution of zOTUs phyla with cosine rhythmicity linked to CFI.

Cosine microbial rhythms were associated with CFI in 96 zOTUs (corresponding to 6.43% of total zOTUs). At 3 months cosine rhythmicity in 9 zOTUs was linked to CFI, increasing to 13 zOTUs at 6 months, and 81 zOTUs at 12 months (Fig. 9A), indicating an increasing number of zOTUs exhibiting cosine rhythmicity interacting with circadian rhythm maturation across the first year of life. At 3 months, most zOTUs linked to CFI were assigned to unknown phyla, while at 6 and 12 months mainly Firmicutes was involved (38.5% and 43.2%, Fig. 9B).

In summary, these results establish associations between circadian maturation in infants, quantified by the Circadian Function Index (CFI), and gut microbial taxa, involving 7.02% of zOTUs. Crucially, microbial composition exhibits diurnal variation, with 1.74% of zOTUs following a sine rhythm, and 6.69% adhering to a cosine rhythm. The expression of both, sine and cosine zOTU rhythmicity, gradually increases across infancy. Finally, the interaction of sine and cosine-fitted microbial rhythm with CFI was found in 1.81% of zOTUs for sine rhythm, and 6.43% of zOTUs for cosine rhythm, the latter experiencing a gradual strengthening with age.

## Discussion

In this longitudinal study across the first year of human life we examined the interaction between sleep rhythm maturation (CFI) and diurnal variation of gut microbiota. We demonstrated that CFI increases with age, confirming the measure as a reliable proxy for circadian maturation when using infant ankle actimetry. Interestingly, infant CFI was associated with gut microbial taxa, a link that strengthened with age. Specific Zero-radius Operational Taxonomic Units (zOTUs), primarily from the phyla *Actinobacteria*, *Firmicutes*, and *Bacteroidetes*, manifested rhythmic fluctuations across the day, following distinct sine or cosine patterns in their relative abundance. Age-related augmentation in rhythmicity was predominantly observed in cosine fitting, suggesting a gradual development of microbial diurnal rhythm with bacterial relative abundance peaking around midnight and reaching a nadir around midday. Remarkably, gut microbial rhythm was connected to CFI, underscoring a relationship between host circadian rhythm and gut microbial diurnality. Particularly cosine microbial rhythm maturation was accompanied with CFI. These findings demonstrate that sleep rhythm and gut diurnal profiles are associated in infancy and highlight the potential that infant sleep may interact with microbial dynamics, which may ultimately foster microbial colonization and developmental health.

This study employed CFI computed from ankle actimetry, which revealed a progressive increase in behavioral sleep rhythmicity. This finding aligns with developmental research indicating that sleep-wake patterns mature during the first year towards adult patterns^55^. The progressive increase in CFI from 3 to 12 months highlights a critical developmental window where sleep-wake patterns gradually evolve from fragmented to consolidated forms. This maturation is critical, as sleep quality, timing, and duration are linked to various aspects of developmental health, including cognitive functions like executive control, neural connectivity, and synaptic stability, all of which are crucial for neurodevelopment and cognitive maturation^3,56–58^. Previous research on the development of sleep-wake patterns used actigraphy to objectively assess periods of sleep, capturing various metrics such as total sleep time, sleep efficiency, and wake after sleep onset^8^. Our use of CFI provides a novel perspective on the maturation of infant sleep rhythms and supports CFI as a proxy for pediatric circadian rhythm research.

The findings confirmed the hypothesis that the circadian maturation during the first year of life is associated with gut microbiota composition. While both circadian maturation and the gut microbiome are expected developmental processes, our results provide novel insights into how these biological changes may be particularly linked. Specifically, we found that the number of bacterial taxa associated with CFI increases across infancy, more specifically showing that increased CFI is linked with higher relative abundance of *Bacteroidetes* and *Proteobacteria* and decreased abundance of *Firmicutes*. These results add to the evidence that circadian rhythm is associated with microbial colonization and the reciprocal interaction between gut microbiota rhythms and the host’s sleep pattern^31,34^. Our findings expand on this perspective by demonstrating that the relationship between gut microbiota and host rhythms encompasses not only sleep behavior but also the broader spectrum of activity-rest cycles extending beyond a single 24-hour cycle. This underscores the possibility of daily activity patterns in shaping gut microbiota composition, which likely impacts development. Conversely, bottom-up signals through gut microbiota may influence behavioral rhythmicity, which, in turn, likely affects long-term developmental outcomes^59,60^.

This study observes patterns of diurnal dynamics in the gut microbiota throughout infancy. Using sine and cosine fitting, we identified rhythmicity in specific bacterial taxa (zOTUs), with 26 showing rhythmicity in sine fitting and 100 in cosine fitting. As expected, data confirm that the rhythmicity of gut bacteria becomes more pronounced across increasing age groups, which aligns with recent research highlighting diurnal fluctuations in microbial and metabolic profiles in infants^34^. The increased rhythmicity of gut microbiota with age may be driven by several developmental changes. For instance, the transition from breastfeeding to solid foods significantly impacts gut microbiota composition, potentially increasing the abundance of rhythmic bacterial taxa^61^. Moreover, regular feeding schedules, which tend to become more consistent during the first year of life, are linked to greater microbial diversity, as shown in our previous research^62^. Feeding regularity may not only influence the composition of the gut microbiota but also support its rhythmicity, a relationship previously demonstrated in animal models^15^. The effects of feeding rhythms on microbiota and host health may extend beyond infancy. In adults, studies have shown that meal timing plays a crucial role in aligning internal circadian rhythms, with benefits for metabolic health. For example, restricting meals to daytime has been found to prevent circadian misalignment and reduce glucose intolerance, a finding relevant for conditions like night shift work^63^. Disruption of microbial rhythmicity has also been linked to metabolic health issues in adults, such as type 2 diabetes, suggesting that interventions supporting microbial rhythms could benefit long-term metabolic health^64^. Recognizing the importance of diurnal rhythms in gut microbiota could lead to new clinical and preventive strategies across the lifespan, especially in metabolic and immune health. Targeting microbial rhythmicity through dietary modifications or probiotic interventions may hold promise for supporting health outcomes from infancy through adulthood.

Our findings show that certain bacterial taxa in the gut microbiota likely exhibit daily rhythms in their relative abundance, which align with indicators of circadian maturation in infants. This suggests that microbial taxa may gradually adjust their diurnal patterns, potentially coordinating with the infant’s sleep-wake and activity cycles. As infants’ sleep becomes more structured over the first year, certain microbial taxa accompany this host behavior, notably within the Bacteroidetes and Firmicutes phyla, which show increasing proportions as CFI scores rise. Bacteroidetes and Firmicutes are known contributors to SCFA production, including butyrate, acetate, and propionate, each of which has distinct roles in metabolic and neural pathways. For instance, butyrate, a major SCFA produced by Firmicutes^65^, has been shown to influence sleep architecture in animal models by promoting non-rapid eye movement (NREM) sleep and affecting the regulation of neural circuits involved in sleep^66^. Studies in older adults suggest higher butyrate and propionate to be associated with improved sleep efficiency and longer sleep duration^67^. In the context of our findings, the increased relative abundance of SCFA-producing taxa (e.g., Firmicutes) in infants with higher CFI may indicate that microbial metabolic byproducts support sleep rhythm establishment. The gut-brain axis may mediate the gut microbiota communication with sleep and circadian rhythms, with the vagus nerve and intestinal epithelial cells transmitting microbial signals to the brain^68–70^. Our findings suggest that higher SCFA-producing taxa, particularly within the Firmicutes and Bacteroidetes phyla, are linked to circadian maturation. Targeting these strains through dietary or probiotic interventions may offer a strategy to support sleep development, physiological resilience, and mental health during early life and beyond^71^.

The presence of gut microbiota is essential for the proper development and maintenance of a fully functional blood-brain barrier^72^. The blood-brain barrier has been reported as a regulatory target of sleep^73,74^. Sleep has accordingly been proposed to restore wakefulness-induced increases in the blood-brain barrier permeability, a process modulated by gut microbiota and their downstream signals^75^. Sleep itself is an active and cyclical brain state governed by both homeostatic and circadian mechanisms and orchestrated by both local as well as centralized neuronal circuitries. This complex regulation suggests that sleep likely serves multiple concurrent functions^76^. In relation to the current findings, the emerging synchrony between host circadian rhythms and gut microbial diurnality in early life may thus reflect a deeply conserved developmental coordination. Accordingly, this work opens fertile ground for evolutionary anthropology, with our findings supporting the hypothesis that systemic host-gut rhythmic alignment facilitates the regulation of metabolic, immune, and neurobehavioral systems, thereby promoting infant survival and long-term fitness. Future research should investigate whether perturbations to this synchrony - such as those resulting from cesarean delivery, sleep disruption, or antibiotic exposure - confer measurable developmental costs, and whether such effects may be attenuated in more congruent caregiving environments.

To our knowledge, no published studies have yet explicitly demonstrated age-related increases in CFI in infants, highlighting the need for further validation of this measure as a marker of circadian system maturation. To further elucidate human microbial rhythms and their health implications, future research should investigate environmental context, particularly diet, which is a key driver of gut microbiota composition^77^. For example, high-fat diet is associated with increased Firmicutes and decreased Bacteroidetes, underscoring the effect of dietary patterns on microbial communities^78^. Even though feeding categories were considered, our analysis lacks precise data on individual diet and the timing of solid food introduction. While our analysis used rigorous longitudinal actimetry data, the computation of microbacterial rhythms relies on cross-sectional sampling. Serial stool samples from the same infants, ideally including nighttime collections, would strengthen the assessment of microbial rhythmicity, as our current sampling was biased toward daytime and may have limited rhythm detection. The observed sine and cosine patterns represent trends across the observed period; but denser sampling could validate diurnal fits and uncover additional rhythmic cycles. Interactions between diet, microbial rhythms, and infant sleep patterns remain critical to understanding these complex developmental systems. Future research should identify specific bacterial strains and metabolic activity or products that promote host circadian function, paving the way for targeted pediatric therapies.

In addition, the abundance of human milk oligosaccharides (HMOs) in breast milk critically shapes the maturation of the infant gut microbiota, e.g., reviewed in^79,80^. HMOs act as selective prebiotics promoting a *Bifidobacterium*-rich gut microbiome, leading to the production of short-chain fatty acids, further they support gut barrier integrity, and modulate immune development. For instance, differences in HMO between breast and formula feeding affect microbial and health outcomes like immune function^34,81^. Emerging intervention trials thus target supplementation with pro- or prebiotics to promote microbial and circadian maturation^82–85^. Based on the observed associations between microbial metabolites (e.g., propionate) and sleep or circadian features in infancy^86^, understanding the impact of HMOs and related interventions remains a high priority for future mechanistic and translational research on infant gut and circadian health. Breastmilk also contains melatonin, which is secreted by the mother in a circadian manner^87^, and may contribute to the regulation of infant sleep and gut microbial rhythmicity. Yet research on this pathway remains limited, and the origin and functional role of melatonin detected in infant stool have yet to be elucidated^88,89^.

While our findings demonstrate an association between microbial diurnal variation and sleep-wake maturation, the directionality of this relationship remains unresolved. It is conceivable that emerging behavioral rhythms entrain microbial oscillations, that microbial dynamics influence circadian development, or that both are shaped by shared regulatory processes. Disentangling these possibilities requires methodological approaches that go beyond correlational analyses. Future studies may apply causal inference techniques – such as dynamic Bayesian networks or structural causal models – or, where feasible, implement experimental manipulations to probe underlying mechanisms^85^.

In sum, this study reveals rhythmic dynamics in gut microbiota across infancy and demonstrates the association between infant circadian rhythm and gut rhythmicity, emphasizing the importance of microbial rhythms in pediatric, neuromaturational, and immunological development. These findings suggest a synchrony between microbial and behavioral rhythms in early infancy, though the underlying directionality remains to be determined. Considering gut microbiota dynamics in early life could lead to targeted interventions for at-risk pediatric groups. For instance, dietary modifications aimed at promoting SCFA-producing bacteria or probiotic supplements containing specific bacterial strains associated with circadian support (e.g., certain Firmicutes or Bacteroidetes strains) could potentially enhance sleep regulation and overall developmental health. Tailoring early-life interventions in this way holds promise for managing health risks and supporting optimal growth trajectories through microbial health.

## Data Availability

The raw data supporting the findings of this study are not publicly available due to privacy and ethical restrictions involving minors. De-identified data may be shared upon reasonable request to qualified researchers. Requests should be directed to salome.kurth@unifr.ch and will require ethical approval and a data use agreement in compliance with cantonal ethical guidelines.

## Declarations

### AI-assisted technologies

During the preparation of this work the authors used chatGPT and perplexity to improve language. After using these AI tools, the authors reviewed and edited the content as needed and take full responsibility for the content of the publication.
